# Who Are the People in Your Gayborhood? Understanding Population Change and Cultural Shifts in LGBTQ+ Neighborhoods

**DOI:** 10.1007/978-3-030-66073-4_1

**Published:** 2020-11-30

**Authors:** Daniel Baldwin Hess, Alex Bitterman

**Affiliations:** 17 Department of Architecture and Design, Alfred State University of New York, New York, USA; 18 Department of Urban and Regional Planning, University at Buffalo, Buffalo, NY USA; 19 University at Buffalo, State University of New York, Buffalo, NY USA; 20 Alfred State College, State University of New York, Alfred, NY USA

**Keywords:** Gayborhoods, Gay neighborhoods, LGBTQ+, Queer, Segregation, Sexual minorities, Urban change

## Abstract

Gay neighborhoods, like all neighborhoods, are in a state of continual change. The relevance of gay neighborhoods—originally formed to promote segregation of individuals who identify as sexual minorities—is lately challenged by advances in technology, experiences with pandemics, shifts in generational opinion and social values, increasing acceptance of LGBTQ+ individuals, and (in certain places) increased rights and protections for LGBTQ+ individuals. This confluence of change has created for many people anxiety related to the belief that gay neighborhoods may be dissolving or even disappearing altogether. Seeking to address these concerns, this opening chapter of the book *The Life and Afterlife of Gay Neighborhoods: Renaissance and Resurgence* presents eight important takeaway messages distilled from the chapters in this volume that, taken together, provide an in-depth overview of the formation, maturation, current challenges, and future prospects of LGBTQ+ spaces in urban environments. Findings suggest that shifts in patterns of residence, socialization, and entertainment for LGBTQ+ residents and visitors across metropolitan space have resulted in certain gay neighborhoods becoming less gay while other neighborhoods become more gay. In this time of social change, economic inequities, public health crises, and technological evolution, gay neighborhoods provide a culturally and historically significant template for communities in confronting adversity, fear, and discrimination. At this point in their maturity, gay neighborhoods have reached a plateau in their evolution; from here we pause to consider the current state of gay neighborhoods—and trajectories that might describe their future form—as we contemplate the importance of gay neighborhoods in the ongoing advancement of LGBTQ+ people everywhere. We conclude by observing that while gayborhoods have experienced a certain level of de-gaying, the trend toward viewing gayborhoods as inclusive and gay-friendly places de-emphasizes the self-segregation aspects of gayborhoods that were important to their initial formation; consequently, while gay neighborhoods may become less gay, other neighborhoods may also become more gay.

## Introduction: Beneath the Crowded LGBTQ+ Umbrella

The rainbow-colored LGBTQ + umbrella is broad and encompasses many people underneath it. Shades of the rainbow umbrella denote various identities of individuals: gay, lesbian, bisexual, trans +, queer, questioning, intersex, allies, and others. Though all of these groups live outside the heteronormative mainstream, little else in common is shared among some members of these groups. Apart from identifying as LGBTQ+, a high-income Black female cis-gendered lesbian, for example, in her journey to understand and express her own sexual orientation, may have little in common with a middle-income gay gender-queer Asian male who both may have little in common with a middle-age White gender-nonconforming trans individual quietly exploring bisexuality at mid-life. All, however, may potentially share in the experience of feeling “ othered,” or living *out*side of predominant heteronormative society.

 CNN anchor Anderson Cooper (who identifies as gay) while speaking with presidential candidate Pete Buttigieg (who also identifies as gay) during the U.S. Democratic Presidential Candidates Town Hall in April 2019, reflected that though the LGBTQ + acronym contains many divisions of identity, the groups contained within reflect people who share vastly different experiences. Cooper questioned the value of such a broadly inclusive umbrella and suggested that those who identify as LBGTQ+ are nonetheless united in that they live *out*side what is considered to be the mainstream norm (CNN 2019). Examined in this way, the term “out” may refer metaphorically to exiting the proverbial closet, but may also refer to stepping *out*side of the heteronormative mainstream. Cooper’s observation calls attention to a heteronormative propensity to generically lump all sexual minorities under a broad LGBTQ + umbrella, but further raises the question of what homonormative might look like. Perceived differences between heteronormative and LGBTQ +-normative creates an overgeneralized binary that become especially problematic when researching “gay” neighborhoods.

Over the past five decades or so, LGBTQ + individuals, couples, and families have made their homes in gay enclaves in cities around the globe. Nonconformity is one commonality among the various identities allied under the LGBTQ + umbrella and while life challenges may be different among certain subgroups, members of the LGBTQ + community maintain respect for the relations between the subgroups as a means of self-preservation. For LGBTQ + people, “ gayborhoods” provide spaces for group members to come together and forge collective experiences (Ghaziani 2015b) and to confront shared challenges that LGBTQ + people have faced for many decades (Chauncey 2008; Seidman 2004). Gay neighborhoods embody this struggle and have been closely linked to the nascent days in the fight for LGBTQ + recognition, equality, and civil rights. LGBTQ+ people are not unique in this regard. Many minorities and subgroups form communities, and neighborhoods are the physical manifestation of these communities. Gay neighborhoods cater to and provide safe harbor for LGBTQ+ residents, citizens, and visitors in settings intended to be separated from a judgmental or unaccepting heteronormative public. For people outside of the LGBTQ + community, gay neighborhoods are often perceived as “gay ghettoes” (Levine 1979) that may be curious or fun to visit, although populated by “different” or “weird” people—affectionately “queerdos” (Kane 2020). It is these differences that fuel a grassroots mobilization among LGBTQ+ people to persevere through adversity; gay neighborhoods thus serve as incubators for empowerment and social change and serve as home base for social movements and the fight for equality that ultimately benefits every corner of society.

**Fig. 1.1:**
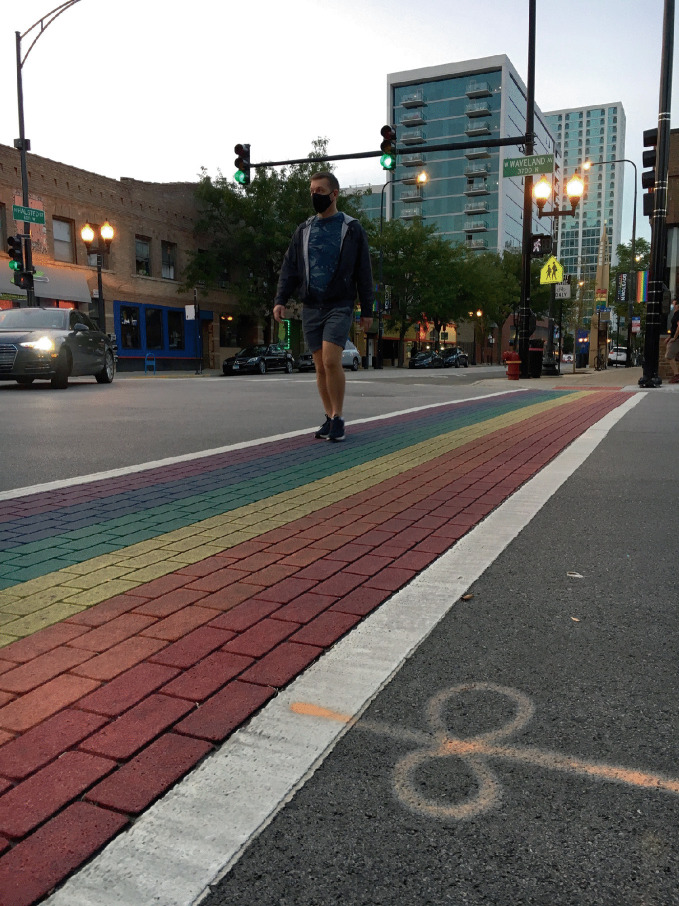
In Chicago and other cities, residents of gay neighborhoods adapt to COVID-19 guidelines including mask wearing and spatial distancing (*Source* Image courtesy of William Ivancic)

Challenges are not unknown to residents of gay neighborhoods. We find ourselves in 2020 in the midst of the COVID-19 pandemic, forty-odd years following the start of the HIV/ AIDS pandemic, and it has re-ignited faintly familiar fears in gay neighborhoods (and beyond) relating to an emerging, mysterious, and deadly contagious disease (see Fig. 1.1). Gay neighborhoods were among the first to experience the HIV/ AIDS pandemic in the 1980s and the disease proved to be both formative and formidable. The unseen gay population was further marginalized and stigmatized during the AIDS pandemic, but residents of gay neighborhoods—along with the broader LGBTQ + community and its allies—rose to the challenge of fighting the deadly pandemic. Gay neighborhoods fostered brave pioneers and some of the very first efforts to assist people with AIDS, to unselfishly raise awareness among the general public about safe sex (when governments were unwilling to do so), and to nurture the value of human life amid profoundly changing circumstances. As a result, gay neighborhoods provide a template of successful place- and community-based adaptation and evolution in maintaining regularity during a pandemic when nothing seems normal. Gay neighborhoods, despite being perceived by some as “other” or “different” can in this way provide much-needed anchors of normalcy and perseverance for broader society.

## Nomenclature: Everyone Belongs

The semantics of “gay” have changed over time and these changes reflect shifts in attitude and shifts in the evolution of mainstream perception. Gay “liberation” during the 1960s evolved into gay “freedom” in the 1970s which evolved into gay “pride” in the 1990s, and this progression was interrupted in the 1980s by the HIV/ AIDS pandemic and the call to power for all LGBTQ + individuals to “ Act Up ” for the right to live free from social stigma. We begin by defining the LGBTQ+ population as consisting of individuals identifying as lesbian, gay, bisexual, transgender and transsexual, queer, questioning, intersex, and other sexual minorities. Individuals self-identify and choose to become members of the LGBTQ + community. The group is inclusive because the community includes members of these sexual minority groups (and their allies), and everyone is welcome to be part. Throughout this chapter and this book we use the acronym LGBTQ+ to signify a broad cultural group (other chapter authors may employ different terminology or acronyms). In editing this volume, we have treated the terms “gay,” “ homosexual,” “ queer” as synonymous and as synonyms for the LGBTQ + acronym as a means to broadly examine the group and its importance along with specific, identifiable urban spaces for sexual minorities. However, we fully acknowledge that the meanings of these words to those in the community differ significantly, and we further recognize the important scholarship about the unique experiences of various sexual minorities (Black et al. 2002; Doan 2007; Gieseking 2020; Hemmings 2002; Nash and Gorman-Murray 2015b; Podmore 2001). It is not our intention to simplify or generalize this complex and diverse group. We understand and acknowledge the imprecision of the LGBTQ+ acronym in that it may make generic the individuals and individualism among its constituent groups and, as noted above, the experiences of LGBTQ + individuals in and among these groups may greatly vary. In this vein, although many gay neighborhoods were historically anchored by a population of gay cis men (Chauncey 2008; Podmore 2021), we consider a “gay” neighborhood to be urban space with some degree of tolerance inclusive of gay men, lesbian women, trans + individuals, intersex individuals, questioning individuals, and various other sexual minorities.

Living among like-minded people, LGBTQ+ residents sought collective security to address their feelings of disenfranchisement and safeguard against oppression manifested in hostility and violence (Lauria and Knopp 1985). In this way, gayborhoods served as refuges from persecution and provided affirming space for marginalized groups. Throughout this chapter and this book, we consider a neighborhood to be a basic building block of a city (Forsyth 2001), and for convenience we interchangeably use the terms “gayborhood,” “ gay neighborhood,” “ gay enclave,” “ gay district,” “ gay village” and “LGBTQ + neighborhood”; we acknowledge the limitations of these labels. We recognize that our decision to use the term “gay” to describe neighborhoods is imprecise because sometimes the term relates to gay men but other times it relates to everyone under the LGBTQ + umbrella (such as when used to denote “gay” pride, which would more accurately be labeled LGBTQ+ pride). Nonetheless, we seek to probe the emergence, evolution, and potential future trajectory of LGBTQ+ spaces in urban environments. It is our sincere hope that over time and with greater study, that these terms can be calibrated and standardized among various disciplines and used in a manner that more accurately captures the individuality of those represented.

## The Other: Refuge and Refusal to Change

For the greater part of the twentieth century, people identifying or classified as LGBTQ + were considered by doctors, police officers, teachers, and other authority figures to be sexually deviant and were often publicly referred to in this way (including labels such as “the degenerates of Greenwich Village” [Duberman 1991]). Perceived sexual deviance was closely associated with dangerous and communicable criminality. The stigma associated with homosexuality remained throughout the twentieth century as authorities openly harassed LGBTQ+ individuals and turned a purposeful or delinquent eye to their rightful protection. Indeed, in many jurisdictions, homosexuality until relatively recently was illegal, and in some places across the globe remains illegal. The anxiety and fear experienced by LGBTQ + individuals as a consequence of this environment of stigma and persecution resulted in a social stigma that kept many LGBTQ+ individuals closeted. Gay neighborhoods emerged over this period as a safe haven for free expression and a respite for all manner of people ostracized or shunned by mainstream society from prosecution, judgement, and violence.

Many gay neighborhoods were seeded in the settlement and movement pattern of sexual minorities beginning in the first half of the twentieth century, and the history of gay neighborhoods is well documented in literature (Chauncey 2008; Ghaziani 2015a; Higgs 1999; Niedt 2021; Orne 2017). The neighborhoods began coalescing in the 1930s, becoming first identifiable in large cities following World War II, but rose to prominence in the 1980s and 1990s partially in response to civil rights struggles and sexual liberation in the 1960s and 1970s and later by the HIV/ AIDS pandemic. During the second half of the twentieth century, recognizable gay neighborhoods emerged in various cities around the world at different times and different rates of settlement. Large urban centers were generally the destination of the “great gay migration” of the post-World War II decades. Original and iconic LGBTQ + neighborhoods—in large cities such as Berlin (Schöneberg), Istanbul (Taksim Square), London (Little Compton Street), Los Angeles ( West Hollywood, which became America’s first gay city), Mexico City ( Zona Rosa), Miami (South Beach), New York (Greenwich Village and Chelsea), Paris (LeMarais), Sydney ( Oxford Street), San Francisco (the Castro), São Paulo (Rua Frei Caneca), Tokyo ( Ni-chōme), Toronto (Church Street), and Washington, DC ( DuPont Circle)—catered mainly to gay men ( lesbians often did not have a notable presence). Each gay neighborhood has its own unique reasons for being and circumstances for development (Gorman-Murray and Nash 2021) and consequently the development and evolution of individual gayborhoods differs.

**Fig. 1.2:**
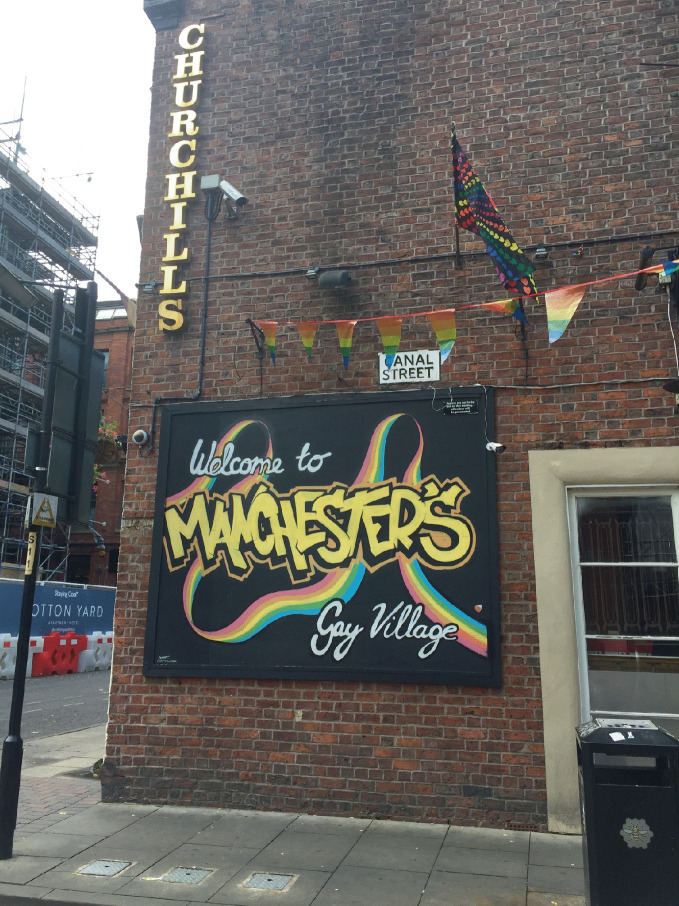
The gay village in Manchester, England, surrounds Canal Street and is one of the largest gay neighborhoods anywhere (*Source* Image courtesy of Daniel Baldwin Hess)

Within large urban centers—perceived as the “natural space” for gays and lesbians (Higgs 1999)—opportunities in gay neighborhoods for leisure and socialization brought together the formative elements for the development of community. Gay neighborhoods have provided individuals with opportunities to develop social networks, to date, and to form relationships (Aldrich 2004; Weinke et al. 2021) and gayborhoods became the center point of social events including gay-themed parties, dances, parades , and street fairs (Bruce 2016; Stone 2021). All of these events helped LGBTQ+ community members to locate their status outside the mainstream. In this way, LGBTQ + neighborhoods provided a supportive community structure which helped LGBTQ+ individuals to succeed. The social and “party” dimension has always been part of the perception of gay villages, where gay men were assumed to engage in frivolity and promiscuity far from the castigating eye of heteronormative society (see Fig. 1.2). As LGBTQ + neighborhoods began to mature in the 1980s and 1990s, gay villages served a central role in delivering health-supportive services—including HIV prevention and clinics, doctor’s offices, counseling services— related to the AIDS pandemic (Ghaziani 2021) as well as mental health resources (Weinke et al. 2021) and social services for displaced and homeless LGBTQ+ youth shunned or ostracized by families. Later, in the 2000s, these same communities became the organizing centers for supporting same-sex marriage and equality.

Many people identifying as LGBTQ + seek freedom of personal expression, while others seek anonymity in gay neighborhoods, where they can live their lives free of judgement or persecution. Centripetal forces serve as the attractions that draw LGBTQ+ people (and others) toward a gayborhood due to the shared benefits derived from a sense of tolerance and belonging (Doan and Atalay 2021). Surrounded by like-minded others, gay men and lesbian women feel more comfortable on city streets in gayborhoods due to attitudes of acceptance and a sense of comfort and belonging, and LGBTQ + residents and visitors felt more free here compared to other places in cities. Gay neighborhoods and their residents have been widely accepted as significant forces in leading and advocating for positive urban change and have reduced the effects of LGBTQ+  minority status by helping to enhance people’s understanding about sexual minorities (Doan and Higgins 2011; Gorman-Murray and Nash 2021), and LGBTQ + community members—and indeed all of society—can experience an improved quality of life when there is an increased level of tolerance.

 Gay neighborhoods also provided a means of entry for mainstream society to better understand LGBTQ + individuals and LGBTQ+ culture. However, as much as heteronormative society identified gay neighborhoods as different or “other,” gay neighborhoods also became places that inclusively celebrated “the other.” In addition to sexual minorities who lived apart from the mainstream, other alternative groups— hippies, punk rockers, prostitutes —could find a home in gayborhoods (Ross and Sullivan 2012). The influence of gay neighborhoods on popular culture—music, theatre, writing, visual arts—especially in the latter half of the twentieth and early twenty-first centuries is especially notable.

## Marginal to Memorable: The Evolution of Gay Neighborhoods

 Gay neighborhoods have often been located in disused fringe locations or undesirable areas of cities where space was available and real estate and rents were cheap. In these off-the-beaten-path neighborhoods, gay men and lesbian women could establish homes and businesses with less fear of being bothered by others or by the authorities, and LGBTQ+ customers could enjoy service without fear of rejection, persecution, or harassment. Property owners in LGBTQ + neighborhoods renovated buildings and performed various acts of inner-city preservation bringing value to the properties through sweat equity. Gay neighborhood leaders worked to landmark and preserve places significant to LGBTQ + history (Miller and Bitterman 2021). As a result, these gay neighborhoods were usually passed over for large publicly-funded urban renewal projects (Gorman-Murray and Nash 2021), thereby protecting the integrity of the built environment and often sparing these neighborhoods from the urban planning missteps common in the mid- to late-twentieth century (Jacobs 1961). This grassroots-level of active preservation and advocacy spared the architectural integrity of neighborhoods—like the meatpacking district in New York City, the South End in Boston, and countless others—and helped to successful reintegrate these neighborhoods into the urban fabric of today.

**Fig. 1.3:**
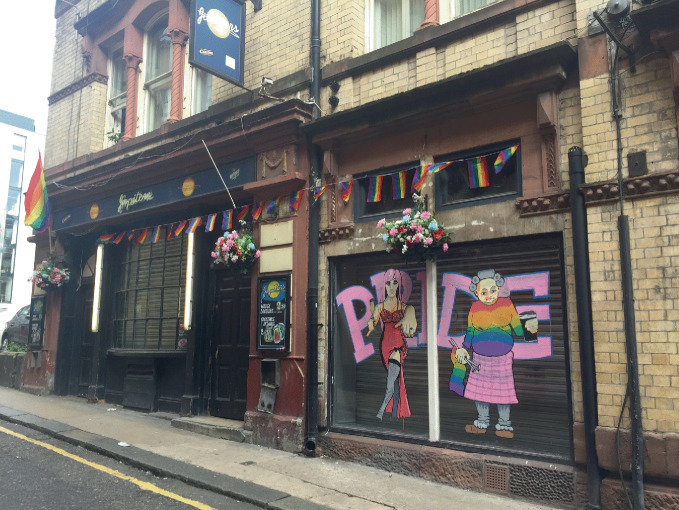
Gay bars are anchor institutions in the Stanley Street Gay Quarter in Liverpool, England (*Source* Image courtesy of Daniel Baldwin Hess)

As understanding and acceptance of LGBTQ + people continued to grow, LGBTQ + neighborhoods often became home to the popular culture vanguard that welcomed, in addition to LGBTQ+ individuals, straight mainstream visitors, bohemian artists, and the cultural *avant garde*. Gay villages cultivated a reputation for restaurants, music scenes, boutiques, and hipster culture (Podmore 2021), thanks to LGBTQ+ pioneers who moved in and settled these places and attracted the pink economy to form around them (Ghaziani 2021). Bars, nightlife, parties, and pride parades became further attractors to gay neighborhoods (see Fig. 1.3). Gay districts in large world cities became tourist destinations, and LGBTQ + neighborhoods flourished “by commodifying the diversity, cosmopolitanism and lifestyle of the inner city” (Nash and Gorman-Murray 2015a, 98). As cultural and economic engines, gay neighborhoods also help to support the vitality of adjacent neighborhoods. In some cities, a “city of neighborhoods” scheme emphasizes gay neighborhoods as cultural anchors that draw tourists, visitors, and residents away from well-known areas like city centers (Gorman-Murray and Nash 2021).

While gay neighborhoods first emerged as marginal outposts, many have transformed (and gentrified) in the last few decades to become universally sought-after districts. Nearly two decades ago, Richard Florida (2004) published a study of “creative places”—locales having the power to attract economic development and foster urban vibrancy—suggesting that concentrations of LGBTQ + residents form the center of an educated and creative community, contributing to the development of local amenities and increases in property values.

### The First Great Plateau

**Fig. 1.4:**
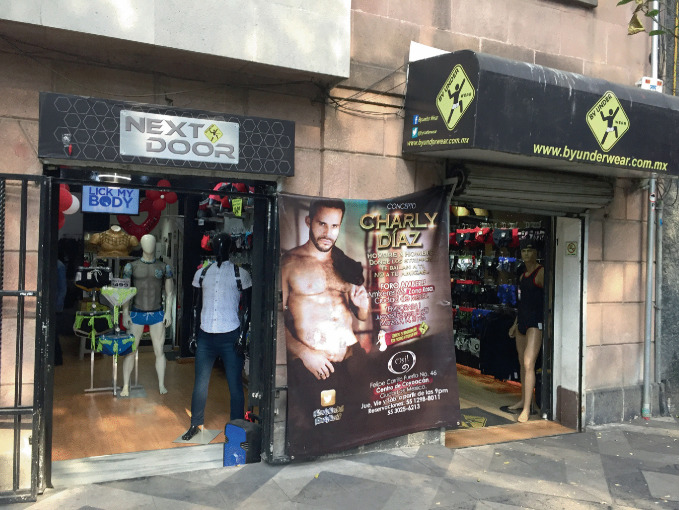
The Zona Rosa (“Pink Zone” in English) is located near the historic center of Mexico City and features retail outlets and nightlife venues amid a gay community (*Source* Image courtesy of Daniel Baldwin Hess)

Over the last two decades many historically gay neighborhoods—such as the Castro in San Francisco and West Hollywood in Los Angeles—have experienced significant demographic change (Bitterman 2020; Hess 2019; Spring 2021; Weinke et al. 2021). Soaring property taxes, rents, and property values—ingredients for hypergentrification—have driven many sexual minorities away from these areas, while many affluent straight professionals and their families have moved into replace them (Christafore and Leguizamon 2018; Ghaziani 2014). With a rise in property values, more affluent people relocate to gay districts and low- and middle-income people have been pushed away (Moss 2017; Zukin 1998). Increases in the number of condominium dwellers are notable, as non-LGBTQ + residents are attracted by neighborhood amenities and the carefree cachet of hip urban living, triggering centrifugal forces that push people away from gayborhoods (Doan and Atalay 2021). Since 2000, a process of “ de-gaying,” during which non-LGBTQ+ people were attracted to gayborhoods (either for entertainment or as residential space), many gayborhoods lost “anchor” institutions, epitomized by the large-scale closure of gay bookstores and gay bars (Eeckhout et al. 2021; Mattson 2019). Neighborhood commercial strips in gayborhoods have been replaced by nightlife venues intended to attract mixed or straight crowds (see Fig. 1.4). As a result, the pink economy has changed significantly (Ghaziani 2021) suggesting a slow erasure of LGBTQ + culture in gay neighborhoods. The closure of iconic gay meeting places, given their importance in sexual minty communities, was often a “turning point” in the decline of gay villages (Doan and Atalay 2021).

Established gay neighborhoods now embody a virtual dimension for LGBTQ + connection (Miles 2021), perhaps redefining the importance of physical place. Compared to mainstream heteronormative communities, LGBTQ+ communities fostered early adoption of technology as a means to augment/enhance physical communities. This newfound reliance on digital technology in gay neighborhoods (largely as a means to take advantage of propinquity) has become increasingly common. Compared to other types of neighborhoods, these digital connections may provide one potential avenue for future sustenance of gay neighborhoods. However, with this shift, some anxiety has arisen among the denizens of LGBTQ + neighborhoods about the perceived demise the incidental physical importance of these spaces which may have interrupted the continuity among LGBTQ+ generational cohorts and accentuated disconnects between various groups under the LGBTQ + umbrella (Bitterman and Hess 2021). The closure of gay bars, emerging virtual gay spaces, generational disconnect, and changes in the character of gay neighborhoods are reminders that as these places transition from being home to generations rooted in struggle to playgrounds of generations benefitting from that struggle, now may be a germane time to examine the present plateau in the trajectory of gay neighborhoods.

## Empirical Plan for This Book

Drawing on a tradition of scholarship about the spatial basis of LGBTQ+ identity (Binnie and Valentine 1999), this book explores perspectives about the past, current, and future conditions of gay districts in cities as a means to better understand the ongoing evolution of gay neighborhoods. We begin by clarifying the role of gayborhoods—home to constituent members of the LGBTQ + rainbow—as places that celebrated “the other” and became the site of sexual liberation from the 1970s to the 1990s (Castells 1983). We are motivated to explore the current plateau in the evolution of gay neighborhoods. We also wish to explore whether gay neighborhoods are declining or are simply evolving, and—in an age of digital connectivity that replaces person connection—the comfort LGBTQ+ individuals experience living as part the heteronormative mainstream. As the stigma associated with LGBTQ + groups decreases, there are changes in people’s needs and desires for living in gay districts (places that initially promoted isolation over integration).

Like all neighborhoods, gay neighborhoods and the dynamics that shape them are unique. This book addresses questions related to the necessity and demand for gay neighborhoods in the future as LGBTQ + people become more accepted as part of mainstream communities. We expect to see new types of gay communities emerge in the future, especially as the baby boom generation and Generation X (and subsequent generations) age into retirement (Hess 2019; Bitterman and Hess 2021), however, these neighborhoods may be different than those we know today. The local, national, and global upheaval related to the COVID-19 pandemic will likely change how people live in and perceive urban neighborhoods, perhaps instigating further—and at present unknowable—transformation to gayborhoods.

**Fig. 1.5:**
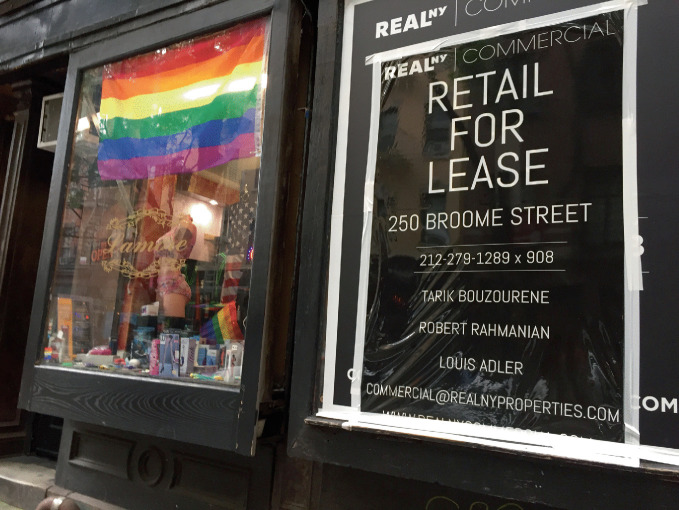
An advertisement for a property leasing opportunity on Christopher Street in New York City’s Greenwich Village (*Source* Image courtesy of Daniel Baldwin Hess)

While recent books have provided various perspectives on the development, growth, and change of gay neighborhoods (Notaro 2020; Ryan 2020; Crawford-Lackey and Springate 2020; Martel et al. 2018; Doan 2015; Ghaziani 2014) and the changing sexual space of cities (Khubchandani 2020; Nagourney 2019; Contreras 2019; Elledge 2018; Evanosky et al. 2018; Orne 2017; Potts 2016; Shaw 2015; Giraud 2014; Murray 2014), this book provides an in-depth exploration of social and cultural phenomena related to the past, present, and future of gay districts. Just as the LGBTQ + community continues to grow and evolve, so too have gay neighborhoods and gay places continued to grow and evolve. Consequently, chapters within the book give special attention to two phenomena in particular: (1) the forces of gentrification that have changed the character of gay districts during the last two decades (Hess 2019; Bitterman 2020), pushing out long-time gay and lesbian residents as the number of non-LGBTQ + residents and visitors increases; and (2) the changing views toward gayborhoods of successive generations of LGBTQ+ residents, with generational-attitudinal perspectives as a significant factor in changing demand among LGTBQ+ groups for gayborhoods (see Fig. 1.5). We believe that the interrelation of these factors both shapes and reshapes the lived experience for LGBTQ + people in neighborhoods and cities. As the stigma associated with membership in groups under the LGBTQ+ umbrella decreases universally, the need/desire for living in places underscored by segregation and self-isolation may change in parallel. As gay neighborhoods continue to evolve, one significant and important risk to note is that the importance of gayborhoods in the struggle for LGBTQ + recognition and rights may be forgotten or erased.

### A Note Regarding Limitations

The geographical reach of the chapters herein is broad, since phenomena relating to the development, maturation, and life cycle of gay neighborhoods is not uniform from country to country or even from city to city (Gorman-Murray and Nash 2021; Doan and Atalay 2021; Bitterman and Hess 2021). This is due to (among other reasons) incomplete and inconsistent collection of data about LGBTQ + individuals and couples (Spring 2021; Frisch 2021) and differing laws and legal protections for LGBTQ + individuals from place to place. Accuracy of broadly applicable assessments regarding “the emergence of” or “the death of” gay neighborhoods is challenging given these legal, social, and economic landscapes and what may be happening in one gay neighborhood may not parallel what is happening in another.

We acknowledge the various limitations that this edited volume presents. Chief among these is an under-representation of scholarship from countries and cities in the Global South (Brown et al. 2010). LGBTQ + civil rights in many countries across the Global South, parts of Asia, and former Soviet republics are less secure than in countries in the Global North and the West. For this reason, some researchers and scholars from countries in the Global South are unable to conduct research or publish works about LGBTQ + issues and communities without significant risk to their professional careers or their personal safety. It is our sincere hope that by making this book broadly available, we can create and support future opportunities for researchers, policymakers, and advocates committed to understanding and evaluating civil rights movements for LGBTQ + individuals across the Global South. While humbly acknowledging the geographic limits presented in this volume, we hope that our contributions in this volume to LGBTQ+ scholarship can help further the geographic reach of this research and support future research as voices in commonly underrepresented areas bravely emerge. Support of this volume from colleagues across the Global South is an important formative step toward increasing global awareness, recognition, and societal equality for all LGBTQ + individuals. Similarly, space consideration in this volume prevented us from giving full attention to LGBTQ+  communities in non-metropolitan spaces (Binnie 2014; Tongson 2011) but our hope is that the research presented here can provide a springboard for others engaging in future research in locales not fully represented in this book.

## Takeaway Messages

The chapters in this volume are constructed in an effort to provide a snapshot of the state of gay neighborhoods in 2021 and beyond. We next offer the following eight synthetic takeaway messages, distilled from the seventeen chapters in this book.

**Takeaway message 1.** **Gay neighborhoods** **are inclusive and are not only for gay men**.

*The term “gay” as a shorthand descriptor in the label for “* *gay neighborhoods” effectively ignores the multipolar diversity among the LGBTQ* + *population (as noted in the “nomenclature” section above). The array of groups represented under the LGBTQ* + *banner may share similar journeys but collectively each subgroup has unique challenges not commonly shared among other sectors of the broader LGBTQ* + *community. Therefore, the term “gay neighborhood” may unintentionally suggest exclusive focus on one specific group*—*gay men*—*and not fully reflect the entire inclusive LGBTQ* + *rainbow.*

- Established largely by gay men, the first gay neighborhoods over time became increasingly defined by inclusivity especially through tolerance of—and kinship with—other sexual minorities, the artistic and creative avant garde, and affluent straight urban professionals. However, these spaces were often viewed by other members of the LGBTQ + community predominantly as gay male space, and as a result of their gay male origins many lesbian women, bisexual individuals, and trans + individuals are consequently less likely to feel a resonant connection with gay neighborhoods. “Gay” male influence in the establishment of gay neighborhoods is still prevalent, but not exclusive.
- The attraction between gay neighborhoods and cultural trendsetters continues today. The distinction between gay neighborhoods and hipster neighborhoods becomes increasingly less clear in neighborhoods recognized for their high shares of sexual minorities (Podmore 2021) and sexual fluidity among younger generations shifts the generational perspective of gay neighborhoods (Bitterman and Hess 2021). As these emerging generational trends become increasingly normalized, the notion of gay neighborhoods demarcated by geographic boundaries may become more challenging for scholars to effectively measure and less relevant to those interested in living there. As Podmore observes (2021, 303): “because the sexual identity of hipster men was ambiguous, their presence could evacuate the area of the hegemonic norms of masculinity that might exist elsewhere.”

**Takeaway message 2.** **Gay neighborhoods** **matter**.

*Gay neighborhoods matter to everyone and are important*—*both historically and currently*—*to the functioning of contemporary* *urban* *culture;* *gay neighborhoods* *support the health and well*-*being of both LGBTQ* + *individuals as well as* *mainstream* *society.*

- Gay neighborhoods emerged in the 1950s and 1960s in large cities as a respite from the critical and shunning eye of mainstream society and overt harassment by authorities. The natural tendency to surround oneself with similar people who share common experiences—known as homophily—underscores the fundamental attraction toward gay neighborhoods (McPherson et al. 2001). Often located in disused urban space, early gay neighborhoods emerged from bohemian enclaves which served as nexuses for a fledgling gay culture that was equated in mainstream society with criminality and deviance. Initially, gay neighborhoods provided a degree of protection from police harassment (safety in numbers) in peripheral urban spaces outside of the public eye. LGBTQ + neighborhoods also give people who identify as sexual minorities a feeling of safety—due to the a perceived feeling of acceptance—compared to other places throughout a city where tolerance for LGBTQ+ individuals may be lesser. Gay neighborhoods provide positive benefits. Living among other LGBTQ + people, gay neighborhoods help fulfill the human desire to build community and capacity for self-actualization, since those who live in areas with higher densities of sexual minorities have lower rates of depression symptoms and higher levels of self-esteem (Weinke et al. 2021).
- Gayborhoods help raise the visibility and advance the cause of sexual minorities under the LGBTQ + umbrella (though at differing rates for each of the various groups). Clustering in certain neighborhoods, LGBTQ + people have raised their visibility and have formed (largely liberal or progressive) voting blocs that help achieve political and social gains. In addition to political functions of voting and elections, Ghaziani (2021) identifies other reasons that gay neighborhoods matter, including providing space to build community and nurture relationships, promoting the pink economy, and supporting political action and activism (Bitterman and Hess 2016b). Most LGBTQ + neighborhoods develop formal and informal support services to improve life quality for all. Without gayborhoods, LGBTQ+ people risk becoming marginalized and under threat of possibly losing rights and liberties they have fought to win (Ghaziani 2021). Over time, the importance of gay neighborhoods solidified as they became the nexus of—at first—the struggle for LGBTQ + civil rights. However, as the HIV/ AIDS pandemic emerged in the 1980s, gay neighborhoods became important centers in the fight against the disease, against ignorance, and against stigma due to illness. Gay neighborhoods later served as the organizational center for pride events which helped to introduce gay life to mainstream culture and established the conditions that eventually made way for legalizing same-sex marriage .
- Gay neighborhoods remain the physical monument to decades of struggle, oppression, and violence. In more recent years, challenges and milestones have been celebrated through LGBTQ+ archives, museums, and exhibits in gayborhoods that educate younger generations about past efforts to secure equality and rights and violence against LGBTQ + individuals (Miller and Bitterman 2021). Gay neighborhoods, throughout each of these eras, have largely provided a welcome and accepting urban space for sexual minorities, LGBTQ+ singles, couples, and families who choose to live there or visit.

**Takeaway message 3. Gay neighborhoods are becoming** **less gay**.

*The trend toward inclusivity may be “* *de*-*gaying”* *gay neighborhoods. As formerly exclusive gay neighborhoods (and gay places within them) have broadened to include “gay friendly,” many gay neighborhoods have attracted* *straight* *people as residents and* *visitors, a phenomenon that dilutes the exclusivity and collective safety offered by* *gay neighborhoods. Along with broader societal forces and greater* *mainstream* *acceptance, heteronormatizing* *gayborhoods* *has diminished the need for LGBTQ* + *individuals to retreat to or self*-*segregate into gay spaces.*

**Fig. 1.6:**
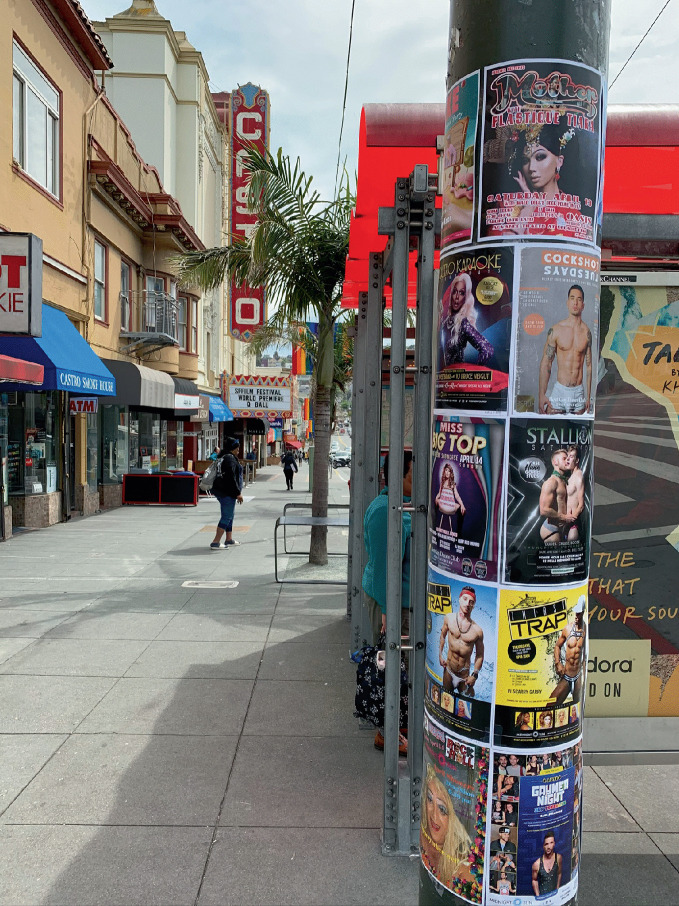
Banners depicting the rainbow flag adorn streetlamp in the Castro in San Francisco (*Source* Image courtesy of Daniel Baldwin Hess)
- Following legislative and social advances—including human rights protections, civil rights, and same-sex marriage —LGBTQ + people have over recent years become increasingly more visible across a variety of locations and are less likely to be confined to or concentrated in gay neighborhoods (Gorman-Murray and Nash 2021). However, just as compelling as the places LGBTQ+ people choose to *live* is where LGBTQ + individuals *do not* live. For example, few gay couples live in the suburbs: “quintessentially suburban neighborhoods have remained closed-off to male same-sex partners, even within a larger MSA context of declining segregation” (Spring 2021, 51). The most inaccessible places for some male same-sex couples, for example, include economically vibrant, “child-friendly,” mostly suburban neighborhoods where they may feel unwelcome or not accepted (Spring 2021), which demonstrates difference in essential requirements for different groups under the LGBTQ+ umbrella to create gay neighborhoods.
- Moreover, recent demographic research suggests that many individuals residing today in neighborhoods with high concentrations of sexual minorities do not themselves identify as sexual minority (Spring 2021; Weinke et al. 2021) and non-minority heterosexuals constitute the majority (Carpiano et al. 2011). Repositioning gay villages as the nexus of LGBTQ + or queer urban space addresses the criticism that gayborhoods are welcoming mostly to gay men and to a lesser extent, lesbians, and even less to queer people who are not out, questioning, or do not identify as either gay or lesbian (Wolf 1979). As the inclusivity of the LGBTQ+ umbrella encompasses more difference, the term “gay” becomes increasingly generalized and its meaning diluted. In this way, the term “gay” is used as a generic shorthand for all LGBTQ + people, which potentially leads to “diversification of the term to the point of meaningless homogenization” (Bitterman 2020, 100). That is, as the LGBTQ+ umbrella has expanded to encompass more diverse groups, the relative life experience of members of LGBTQ + subgroups may be less comparable and less interconnected especially when overlaid by other understandings and complexities related to diversity. The effects of this hyper-inclusivity may result in an unintended dilution of gay neighborhoods by “ de-gaying” the very neighborhoods meant to protect and empower LGBTQ + people.
- During the 1990s and 2000s, a dramatic decline occurred in the number of gay bars in gayborhoods (Eeckhout et al. 2021; Mattson 2019). See Fig. 1.7. An increased demand for larger venues for staging expansive organized parties reduced the demand for smaller neighborhood bars, and gay-friendly mega clubs offered opportunities for more entertainment spectacle in mixed parties. This loss of regular neighborhood bars has reduced opportunities for social mixing among LGBTQ+ people from various generations (Bitterman and Hess 2021; Eeckhout et al. 2021). While previous generations of gay men preferred to socialize in bars visited strictly by gay men, those attending parties in gay neighborhoods today seek inclusive “gay friendly” dances and events (Eeckhout et al. 2021): “the relatively exclusive, niche-specific, semi-public spaces of lesbian and gay bars that promised a safe haven in a largely hostile environment lost their raison d’être faster than anyone would have expected a few decades ago” (Eeckhout et al. 2021, 238). These changes in how LGBTQ + individuals socialize in gay neighborhoods underscores broader societal shifts among younger generations (Bitterman and Hess 2021).

- As they matured, gay neighborhoods transitioned from destinations primarily for socialization (in bars, restaurants, cafes, and bookstores) to places for residence, where LGBTQ + people established their homes and built community (Niedt 2021). More recently, as gayborhoods gentrify, heterosexual people have moved in and gay neighborhoods have become attractive mixed-use residential neighborhoods containing amenities with broad appeal and progressive cachet. The conventional concept of a gay neighborhood (a “village” with a mix of everyday services, modeled on Greenwich Village in New York City) is being replaced through demographic shifts by “emerging” LGBTQ+ places in urban-metropolitan space (Bitterman 2020; Hess 2019). The emergence of gay neighborhoods in other settings reflects a redefinition of what is important in residential environments and surrounding communities for LGBTQ + individuals and same-sex couples, resulting in “a new normal” for gay neighborhoods. These “emerging” places likely contain neighborhood services and amenities that have not in the past been strongly associated with gayborhoods.
- Examples of the “ de-gaying” and the evolution of LGBTQ+ neighborhoods are plentiful. Across Atlanta, this phenomenon produces an outward centrifugal force that redistributes LGBTQ + residents from gayborhoods to other places (Doan and Atalay 2021). This dispersal is evident by the decentralized display of symbols associated with gay pride and gay neighborhoods (some of which are shown in Fig. 1.6)—pink triangles, rainbow flags, and equality symbols—that are dispersing across metropolitan space and becoming more ubiquitous. This “rainbow diaspora” has produced a measurable increase in the visual display of the rainbow flag in neighborhoods in Toronto—diffused from the historically gay Church-Wellesley neighborhood— into the Parkdale and Roncesvalles neighborhoods and across the city (Bitterman 2021). These integrative examples suggests both greater acceptance of LGBTQ + individuals and greater dispersion of LGBTQ+ individuals from specific gayborhoods.

**Fig. 1.7:**
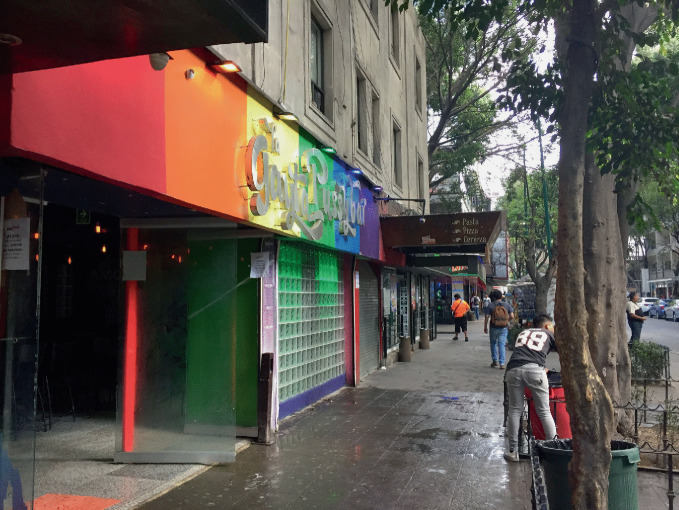
Bars and nightclubs are prominent in the Zona Rosa (“Pink Zone” in English) in Mexico City (*Source* Image courtesy of Daniel Baldwin Hess)
- Among those traditionally not found beneath the LGBTQ + umbrella, gender fluidity and diversity of gender expression—long conflated with homosexuality and “being gay”—has become more clearly articulated and is becoming more socially accepted. Shifting perceptions of gender, gender identity and fluidity, and gender expression—paralleling the rise of “gay friendly” culture—have given a broader mainstream voice to queer culture (Seidman 1994). Amalgamations of words that reference homosexuality as a cultural touchpoint are becoming increasingly common. For example, “ metrosexual”—a straight male with grooming habits or fashion-conscious proclivities typically associated with gay men—is one example of this cross-over. Similarly, a “ lumbersexual”, is a homosexual with certain “butch” characteristics (manner or dress) reminiscent of a lumberjack. “ Cuomosexuals” are those individuals who appreciate the efforts of New York State Governor Andrew Cuomo, especially in fighting the COVID-19 pandemic (Miles et al. 2021). In contrast to the “ de-gaying” of gay neighborhoods, this shift could be considered the “gaying” of heteronormative society. Observing the more recent blurring of differentiation between queer culture and hipster culture in the gay village of Montréal, Podmore (2021, 303) argues that “the boundaries between hipsters and queers were blurred rendering all young people in Mile-End as queer.”
- Many LGBTQ + individuals today—especially younger groups—embrace a broadly inclusive definition of sexual orientation and find little value in labels such as “gay,” “ lesbian,” “transgender,” and other sexual minority groups (Podmore 2021). These younger individuals may view gayborhoods as relics of the past, or may find gay neighborhoods not to be welcoming in ways that match contemporary sensitivities toward inclusivity (Bitterman and Hess 2021). In the same way, the older cohort in gayborhoods is often less comfortable with the sexual diversity that younger people easily accept nor the sexual fluidity they may practice. It can be difficult to distinguish between queer and hipster, and the hipster aesthetic marks gayborhoods as distinctly non-heteronormative space. For non-LGBTQ + individuals, “the idea that sharing space with hipsters serves to disrupt heterosexual norms and to recode the spaces as progressive, creative and open” (Podmore 2021, 304). This is a reminder that we now live in a post-binary multipolar world (Hess 2019) and this change is reflected in neighborhoods and places. These social shifts represent significant changes in thinking and perspective underpinning generational change. A tendency for younger groups to embrace less prescriptive and defined gender and sexual orientation will likely impact gay neighborhoods and indeed all neighborhoods (Bitterman and Hess 2021).
- The “ de-gaying” of gay neighborhoods has elevated their visibility but also their vulnerability. Gay neighborhoods, as places favored by LGBTQ + people to visit for entertainment and socialization and to reside in, also provide space for those who do not identify as sexual minorities. Research by Nash and Gorman-Murray (2014) suggests that rather than understanding changing gendered and sexual landscapes as manifestations of decline, it is more suitable to understand gayborhoods as part of relational geographies between neighborhoods supporting visible queer populations and marking greater social cohesion. Groups of people may now visit gayborhoods who would not have done so when gayborhoods were seen strictly as playgrounds for gay men.
- Many researchers have investigated the impact of gentrification on gay villages including the displacement of LGBTQ + commerce and households and the “de-gaying” or the loss of LGBTQ + consumers and the integration of the broader public into local markets (Doan and Higgins 2011; Ghaziani 2014; Gorman-Murray and Nash 2016; Ruting 2008). We conclude this section by observing that while gayborhoods have experienced a certain level of de-gaying, the trend toward viewing gayborhoods as inclusive and gay-friendly places de-emphasizes the self-segregation aspects of gayborhoods that were important to their initial formation (Moss 2017); while gay neighborhoods become less gay, other neighborhoods become more gay.

**Takeaway message 4. Virtual connections enhance** **gay neighborhoods**.

*Contrary to the perception that technological change*—*online presence and virtual connection through* *social media* *(dating and hook*-*up apps)*—*has hastened the* *decline* *of gayborhoods by reducing the need for physical presence, we argue that* *technology* *enhances rather than replaces the social aspects of* *gay neighborhoods*.

- During the last decade a broad proliferation of location-based smartphone dating and hook-up apps including Grindr, FindHrr, Scruff, and others have replaced Internet dating websites from the 1990s and 2000s, such as Adam to Adam and Planet Romeo. Unlike online dating sites and newspaper personal ads before them, these apps offer geocoding that serves to “decenter placemaking efforts” (Ghaziani 2021, 89). Consequently, remarkable changes may be looming:
-
  … LGBTIQ life has been transformed by the virtualization of sexual networks in urban space as a result of new technologies. Digital, mobile, and social media allow for instantaneous contact across the globe, allowing LGBTIQs to connect across geographical boundaries beyond their immediate (urban) dwelling. At the same time, location-based services, in particular dating apps such as Grindr, allow LGBTIQs to identify and connect with other LGBTIQs within their urban or even rural contexts. (Eeckhout et al. 2021, 239)
- Technology, as a consequence, may transform certain functional aspects of gay neighborhoods and render physical proximity less relevant because physical aspects of gay neighborhoods now have virtual dimensions for LGBTQ + connection. The centrifugal pull away from gay neighborhoods may shift as a result (Doan and Atalay 2021), because location within in a gay neighborhood or even in the same city or country is unnecessary to use hook-up apps to find others. Today, most everyone can be connected digitally, since gay dating and hook-up apps transform “any street, park, bar or home into a queer space by brokering a meeting between mutually attracted individuals” (Miles 2021, 207). In this way, any physical locale can acquire a queer overtone when it is employed as a meeting place relating to LGBTQ + online/virtual connection (Miles 2021), and technology is used creatively by LGBTQ+ people as they inhabit gay spaces other than gayborhoods (Wu and Ward 2018). In this way, gay neighborhoods could emerge as neutral and safe “meeting grounds” for hookups and dating. For Miles (2021), this creates in gayborhoods a “ hybrid reality” formed from layered physical place and digital space. Consequently, a gay neighborhood can be created anyplace, enabled by “pre-screening” of people and places in social apps.
- Online environments and apps may perhaps facilitate the decline of gay neighborhoods, permitting LGBTQ + people to scatter from gay villages to new residential settings across metropolitan space: “queer dating and hook-up apps are variously blamed for destroying gay neighborhoods and celebrated for reinvigorating them; dismissed as impediments to queer community by some and hypothesized by others as virtual sites for new and often liberatory communities of their own” (Miles 2021, 210). Smartphone apps, in this way, could be credited as a leading factor in LGBTQ + deconcentration from gayborhoods. Certainly, the ability to connect with others for sex and dating lessens the centrality of the former go-to gay neighborhoods and venues—shops, bars, restaurants, bookstores, community centers—within them.
- We argue, however, that while online apps *enhance* physical space in gayborhoods, they provide an *overlay* upon lived physical space but do not *replace* the lived city. In other words, technology overlaps but does not replace propinquity and physical presence. Although LGBTQ + life “has been transformed by the virtualization of sexual networks in urban space as a result of new technologies” (Eeckhout et al. 2021, 238), the importance of place in gayborhoods is not threatened with erasure solely because of changes in the way LGBTQ + people use or engage technology.
- During the early days of the HIV/ AIDS pandemic, gay neighborhoods served as ground zero for LGBTQ + activists to organize and demand change. We emphasize the importance of neighborhoods, yet virtual connections for LGBTQ+  community members can transcend neighborhoods and go anywhere—both physically/spatially (global) and temporally. If gayborhoods are indeed in decline as physical spaces, they now—in the Internet age and beyond—have an “electronic afterglow” that is embodied in smartphone apps and reflected in people’s individual and collective digital presence (and the legacy of this presence) (Coffin 2021).
- Digital connectivity has accelerated during the COVID-19 pandemic (Miles et al. 2021). We conclude this section by noting that various generations of LGBTQ + individuals engage technological change differently, and the COVID-19 pandemic has further influenced the way nearly everyone engages technology (Miles 2021). Consequently, we expect that people’s response due to coronavirus-related lockdowns will further shift how LGBTQ + people cope with and embrace technology vis-a-vis the places in which they reside (Miles et al. 2021) and frequent. In this way, gay neighborhoods will likely—stemming from the COVID-19 pandemic—again become engines of change for LGBTQ+  communities and beyond.

**Takeaway message 5. The disappearance of** **gay neighborhoods** **could diminish safe spaces for LGBTQ**+** individuals**.

*The perceived* *decline* *of* *gay neighborhoods* *has produced concern and* *anxiety* *among the LGBTQ* + *population about possible disregard for the original accomplishment of establishing* *gayborhoods* *as safe and inclusive urban space for LGBTQ*+* individuals.*

**Fig. 1.8:**
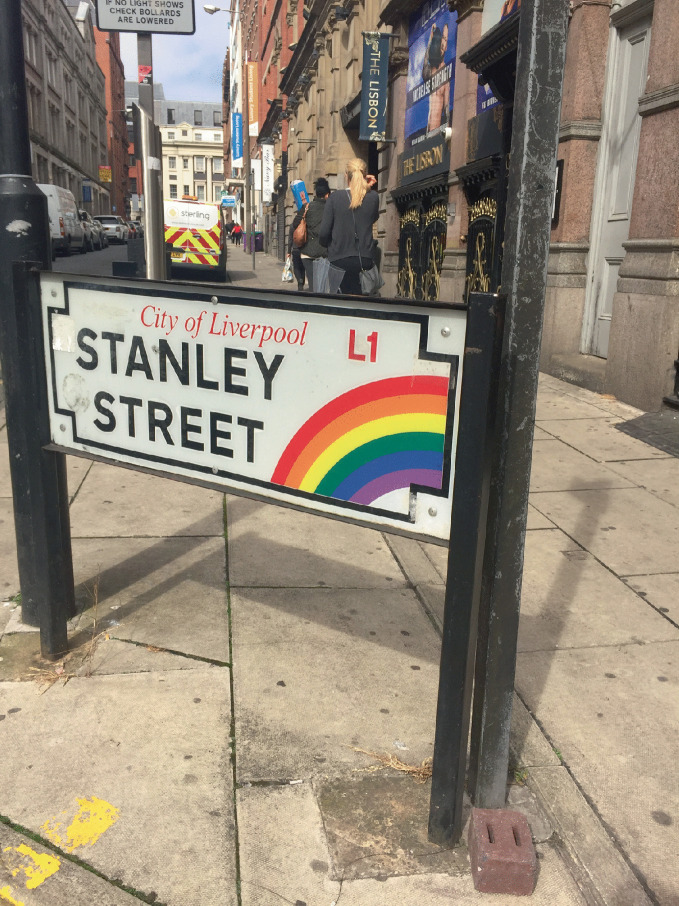
Signage welcomes visitors to the Stanley Street Gay Quarter in Liverpool, England (*Source* Image courtesy of Daniel Baldwin Hess)
- The apparent slow erosion of gay neighborhoods, loss of collective identity, and struggle to preserve historical achievements creates a cultural stress. LGBTQ + people experience stressors over and above the routine stressors that all people encounter, increasing the likelihood that minority group members experience mental health problems (Weinke et al. 2021). LGBTQ+ people have a number of resources available to them—typically in or near gay neighborhoods—to help with “minority coping” related to the “ minority stressors” they experience. In this way, gay neighborhoods provide various supports to LGBTQ + individuals and have a positive impact on the mental health of sexual minority young adults, above and beyond the influence of their individual characteristics (Weinke et al. 2021). Although multiple factors appear to contribute to sexual minorities’ poorer mental health (Weinke et al. 2021), most researchers believe that the stress caused by sexual stigma and prejudice is the most significant factor, and gay neighborhoods can help mitigate this stress across the lifespan, though younger generations not directly participating in the struggle for LGBTQ + civil rights may be unaware of the importance of community that gay neighborhoods provide and support (Bitterman and Hess 2021).

- Gentrification and hypergentrification may slowly edge gay residents and businesses away from gay neighborhoods (Bitterman 2020; Hess 2019; Moss 2017). With closure or displacement of LGBTQ + residences, bars, businesses, and services, the “gayness” of gay neighborhoods can be vulnerable to decline, eventually resulting in destruction or obsolescence that leads to erasure (Eeckhout et al. 2021; Mattson 2019). Anxiety and fear related to this potential erasure exposes the vulnerability that LGBTQ + people experience (Weinke et al. 2021) regarding their comfort with their place in society. Iconic institutions and venues within popular gay neighborhoods— bookstores, bars, nightclubs—are closing, and these place are important to the identity of people in the LGBTQ + community and may even have been part of “coming out” stories. As the LGBTQ+ population share in gay neighborhoods appears to decline—or as the gayborhoods become more “ mainstream” and populated by non-LGBTQ + people—a foreboding sense of potential and monumental loss of LGBTQ+ spaces and culture emerges. Gorman-Murray and Nash (2021, 250) explain that “ anxiety about ( gayborhoods”) possible decline has grown, particularly with the loss of several iconic businesses, rising rents and an influx of heterosexuals into the condominium market and entertainment venues.”
- Older generations of LGBTQ + pioneers helped to build gay neighborhoods as safe spaces unthreatened from the harassment and persecution of a hostile world (Bitterman and Hess 2021). These respites provided fertile ground for an early generation of pioneers to organize, mobilize, and activate a wave of advocacy for LGBTQ+ recognition and rights. These trailblazing generations shifted the public perception of “being gay” away from illegality and dereliction toward tolerance and normalcy. The societal stigma attached to being gay was magnified during the HIV/ AIDS pandemic—and the adversity experienced by gay men during (and after) that pandemic—reshaped and fueled a generation of LGBTQ + activists, pioneers, and allies (Bitterman and Hess 2021). Challenging those in power and the institutions of power was no small effort for these trailblazers. Gay neighborhoods served as the geographic centers of a cross-generational movement, and gay neighborhoods remain important to the shared cultural memory of the struggle for dignity, rights, and civil protections—aspects that undergrad LGBTQ + pride celebrations today—for gay men and lesbian women.
- However, younger generations that did not directly participate in the struggle for LGBTQ + rights may not fully grasp the importance of gay neighborhoods for LGBTQ+ culture and lesbian and gay life (Bitterman and Hess 2021). This may signal a disconnect between older and younger LGBTQ + generations, especially as fluidity in gender expression and sexual orientation shifts LGBTQ+ identity among the younger generations (Bitterman and Hess 2021). As a result, a lack of continuity and awareness may threaten the existence (Podmore 2021) and lasting value of gay neighborhoods (Miller and Bitterman 2021). In the United States, a national effort was started during the Obama administration to identify, memorialize, and landmark sites that provide significance to the history of LGBTQ + community (Miller and Bitterman 2021). This important endeavor was intended to affirm the critical importance and relevance of these sites for generations to come (Bitterman and Hess 2021). The survival of smaller gay districts (and gay districts located in small- and mid-sized cities (Forstie 2008)) is more threatened than established gay districts in larger metropolitan areas with critical mass in LGBTQ+  communities (Ghaziani 2021) and some locations have informally commemorated LGBTQ + significant places within or near gay neighborhoods, as shown in Fig. 1.8.

**Takeaway message 6. Same-sex couples have shifted their residences away from** **gay neighborhoods**.

*Members of the LGBTQ* + *population are shifting their residences or settling in new patterns.* *Gayborhoods* *have consequently experienced noticeable diffusion since 2000, with many LGBTQ* + *couples relocating to other neighborhoods.*

- In 2011 the media began reporting the residential and commercial dispersion from Montréal’s gay village to other parts of the metropolitan area (Podmore 2021). Generally, gay neighborhoods with a declining population of male couples tend to be situated in central cities, where housing values are rising, where median incomes are rising, and with lower home ownership rates than other neighborhoods (Podmore 2021). Chapters throughout this volume explain how gay men and gay male couples historically self-segregated into gay villages, however the trend since 2000 suggests declining self-segregation in gayborhoods with more same-sex couples spread across *all* urban neighborhoods (Spring 2021). As gay people leave gay neighborhoods, the resulting demographic profile of those who remain (combined with newcomers who replace departing gays and lesbians) is *less gay* than it was before (i.e., an overall smaller share of sexual minority identifying people in the population and an overall higher share of non-LGBTQ + individuals). In the United States, census data suggests that certain new gay neighborhoods—mostly in central cities (and not in suburbs)—sprouted between 2000 and 2010, but they were different in character (i.e., more demographically “average”) from established gay neighborhoods (Spring 2021). Mobility data from the U.S. census suggests that the key trend driving declining segregation in gayborhoods is an increase in male same-sex households across other neighborhoods throughout metropolitan space (Spring 2021).
- As members of the LGBTQ + community shift housing locations, evidence of other types of LGBTQ-friendly or inclusive neighborhoods is emerging (Bitterman 2020; Spring 2021). As established gay neighborhoods deconcentrate, clusters of male same-sex couples [and other LGBTQ + couples and individuals] emerge elsewhere, so that the original gayborhoods become less isolated and LGBTQ+ individuals become more integrated. Again, the phenomenon of gay neighborhoods becoming slightly less gay, while other neighborhoods become slightly more gay, becomes evident as LGBTQ + people reconcentrate in other spaces away from gay villages, producing a greater number of gay enclaves while the original gay neighborhoods become less self-segregated.
- Visual assessment evidence in neighborhoods around Toronto indicate the emergence of enclaves of LGBTQ+ people living away from the established gay village (Bitterman 2021), supporting similar observations in Atlanta where greater integration has shifted LGBTQ + life to peripheral parts of the metropolitan region (Doan and Atalay 2021). Importantly, we note, a spatial diffusion of LGBTQ + culture away from gayborhoods does not suggest a complete or pending demise of gay neighborhoods; instead, we argue that gay neighborhoods have arrived at a plateau from which continuous and dynamic re-spatializations across metropolitan space (Coffin 2021) and the memorialization of gay neighborhoods and places within them (Miller and Bitterman 2021) may occur.
- We draw attention in this takeaway message to our important observation that—although the preceding discussion has relied on, among other scholarship, a recent study of LGBTQ + residence patterns using U.S. census data (Spring 2021 ) drawing on previous comparable studies (Gates and Ost 2004)—data collection related to LGBTQ+ individuals, including their presence and activities in gay neighborhoods, is incomplete or is not collected at all. This poses challenges for elected leaders, advocates, and scholars in tracking LGBTQ + individuals, couples, and families. Only certain entities collect data about same-sex partnerships and LGBTQ + individuals. Sometimes the data collected depends on the political predilections of the administration in power. Frisch (2021) reports that the historic record of LGBTQ+ individuals through U.S. census data is troubled and incomplete and has resulted in erasure and marginalization. This discontinuity of information provides challenges for researchers, especially in the Global South and Middle East and in countries where homosexuality remains illegal or stigmatized and where little or no data is collected.
- Certain methodological challenges are also present. For example, the U.S. census relies on census tracts to represent neighborhoods, even though the boundaries of census tracts are arbitrary and do not reflect administrative district or other elements of urban geography (Spring 2021). Consequently, varying physical definitions of neighborhoods could lead to estimates of demographic and geographic phenomenon—including segregation patterns—that are lower or higher than the realities of characteristics in neighborhoods. These inconsistencies amount to de facto discrimination by omission, ignorance, or willful disregard and creates among LGBTQ+ individuals an invisible and indiscernible minority hiding in the shadows of heteronormative life (and reflected in administrative data and datasets). We register concern with regard to these inequities, which may compound as integration of LGBTQ + people continues across urban neighborhoods.

**Takeaway message 7. Gay neighborhoods, at this point in their stage of maturation, have reached a plateau**.

*By 2020,* *gay neighborhoods* *may have reached a plateau in their evolution; from this point in time and space, there are various trajectories into which gay neighborhoods may proceed in the coming years. A plateau, we caution, is an expected part of an evolutionary process and not necessarily a signal that* *gay neighborhoods* *are extinguishing.*

- As people seek to better understand the post-gay, post-binary world in which we find ourselves, there is a recognition that gayborhoods have possibly reached a “pause point” in their evolution. From this position—a plateau or a natural evolutionary stage—there are various trajectories which the future meaning and form of gayborhoods may follow (2020). While a simple linear model can be used to conceptualize the dissolution of gayborhoods when society has eventually reached full acceptance of LGBTQ + and segregation is unneeded and unwanted, we can more realistically imagine much nuance—provided by the addition of complex centrifugal and centripetal forces that entice LGBTQ + people and other population subgroups toward or away from gayborhoods—to the model (Doan and Atalay 2021; Duberman 2018).
- As LGBTQ+ neighborhoods change and evolve, some current or original gayborhoods will be succeeded by or replaced with new LGBTQ + urban space. For example, “micro-communities of LGBT residents will likely arise, constituted perhaps from ten nearby apartments or ten nearby apartment buildings, rather than the size of ten city blocks, as in the past” (Hess 2019, 234). Demographic subpopulations under the LGBTQ+ umbrella, such as older LGBTQ+ adults (Bitterman and Hess 2016a), may settle in gay-friendly apartment complexes or resort-like LGBTQ + retirement centers (Hess 2019). Nuanced re-spatialization—perhaps taking forms that we cannot yet imagine—may describe future gayborhoods (Coffin 2021).
- From a position on this plateau, we pause to contemplate the potential future trajectory of LGBTQ + urban space, and we suggest that it is unwise to fixate on the decline or death of gay neighborhoods but to instead better understand and explore emerging concentrations of LGBTQ+ residents in new formations across metropolitan space, especially other central city neighborhoods that have not long been associated with a LGBTQ + presence but may acquire one. Gay neighborhoods in cities continue to evolve and may reach “stagnation” points on a plateau: “ Oxford Street has continued to decline materially and imaginatively as *the* gay village within Sydney, [ Australia] while Newtown and the inner west have continued to solidify as queer neighborhoods” (Gorman-Murray and Nash 2021, 256). Similarly, the gay village in Montréal has matured from an enclave for gay men to an inclusive space dominated by a queer presence (Podmore 2021). Similar observations have been made with regard to other cities by other authors in this volume.

**Takeaway message 8. The evolution and history of gay neighborhoods is empowering to the LGBTQ** **+** **community**.

*While the future meaning and shape of* *gay neighborhoods* *is unclear, it is important to reflect on the profound and formative effect* *gayborhoods* *had on gay life and LGBTQ* *+* *culture during the 1970s, 1980s, and 1990s. Stemming from this remarkable period of cohesion and maturation, the historic importance of gay neighborhoods will continue to influence the afterlife of LGBTQ*+ *urban* *space. In this way, gay neighborhoods will continue to reflect the struggle for recognition, equality, and* *civil rights* *for* *sexual minorities* *for future generations.*

**Fig. 1.9:**
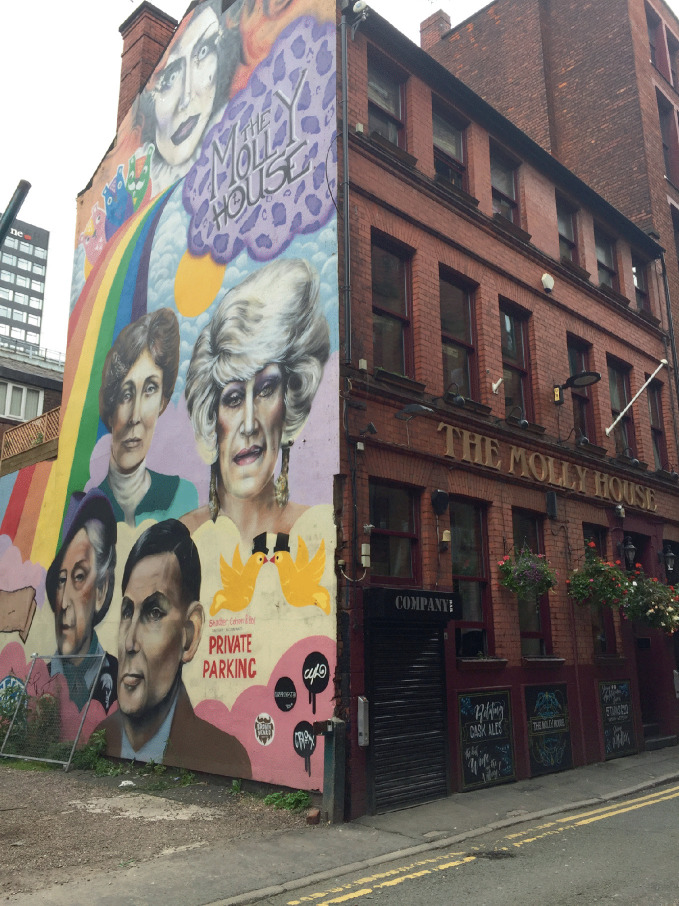
A mural at The Molly House in the Canal District in Manchester, England depicts gay and feminist icons (*Source* Image courtesy of Daniel Baldwin Hess)
- Now, the physical building blocks of gay neighborhoods—commercial establishments ( bars, restaurants, bookstores), services ( community centers, health clinics), and residences—may be removed or displaced due to various urban forces including neighborhood change, revitalization, and gentrification and socio-cultural influences (tastes, preferences, and attitudes) and even equal rights legislation (Bitterman 2020; Eeckhout et al. 2021; Hess 2019). However, if gayborhoods (or elements of gayborhoods) are at risk or indeed disappearing, then the need to preserve these memory spaces becomes urgent to preserve the places and document the memories of residents in the neighborhoods and social action that occurred there (Miller and Bitterman 2021) especially for future generations (Bitterman and Hess 2021). See Fig. 1.10.

- Prior to the twentieth century, a great deal of repression of gay and lesbian life and little acknowledgement occurred of bisexual or trans + life. Various social and cultural forces converged in the second half of the twentieth century, and LGBTQ + neighborhoods were established and grew and became places from which pride for sexual minorities emanated and through which the fight for equal rights for LGBTQ+ individuals was waged and has been (fragmentally) won over time (see Fig. 1.9).

**Fig. 1.10:**
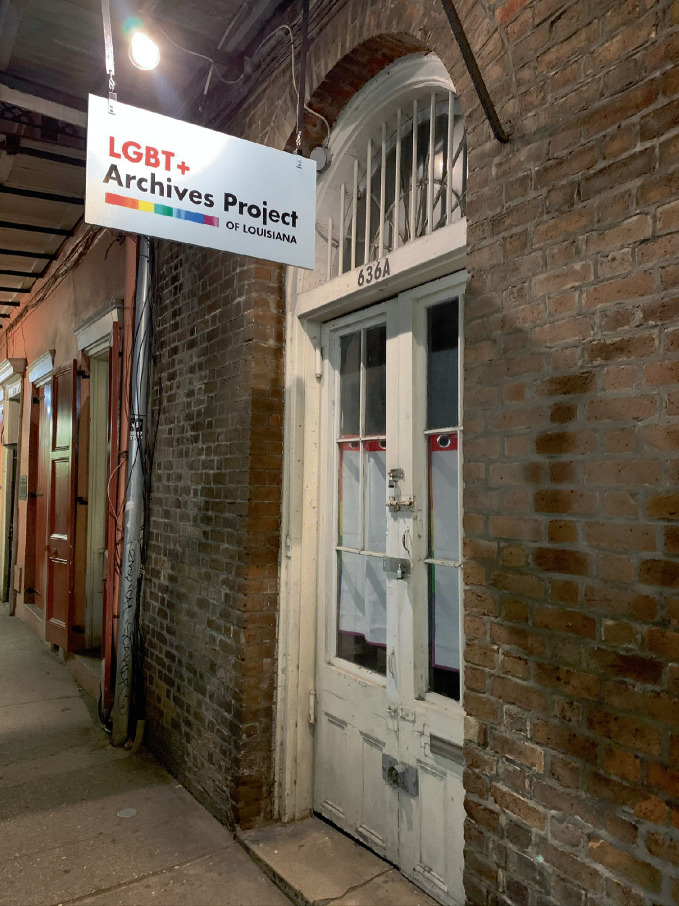
The LGBT+ Archives Project of Louisiana is located just outside the French Quarter in New Orleans (*Source* Image courtesy of Alex Bitterman)
- As we mention in takeaway message 5, gay neighborhoods can be vulnerable to erasure due to urban revitalization and gentrification (Eeckhout et al. 2021). If gay neighborhoods are becoming less gay, declining, or potentially disappearing, it seems that they are being reinforced and even replaced by a diaspora of LGBTQ + cultures across time and space as other neighborhoods become more gay and LGBTQ+ inclusive. Gay neighborhoods possess a legacy and history that has meaning beyond their current physical life; Coffin (2021) explains that “non-climactic gayborhoods leave “ afterglows,” affects that continue to exert geographic effects in the present and near future” (Coffin 2021, 373) and “a gayborhood can have an afterlife even if its physical presence is lost” (Coffin 2021, 381). LGBTQ+ neighborhoods can consequently be expected to continue to “exert an influence, albeit an altered one, on the sociospatial dynamics of urban conurbations (and beyond” (Coffin 2021, 375). The physical presence of LGBTQ+ urban space can thus be replaced by a “subconscious image” of gay neighborhoods (Coffin 2021). In this way, aspects of gayborhoods live on even after aspects of the physical place have been removed:
-
  This is because gayborhoods, like most meaningful places, produce intense affective experiences that leave their marks in the minds and bodies of humans, as well as in the heterogeneous bodies that constitute the nonhuman environment. … If a plateau, such a physical place that can be experienced first-hand, becomes sufficiently intense, such as a highly concentrated gayborhood that forms the heart of local LGBTQ + cultures, then it can leave an afterglow that continues to exert an effect through the bodies of those that experienced this intensity. Put differently, the plateau describes a place as a physical-sensual environment within a particular territory, while the afterglow denotes a post-place as an imaginary-symbolic effect that percolates through deterritorialized networks. (Coffin 2021, 381)

## Conclusion: Resurgence and Renaissance

Throughout the 1990s and 2000s, established gay neighborhoods became increasingly “ less gay,” and more mainstream, while many LGBTQ + residents moved to other neighborhoods and communities (arguably making these neighborhoods “ more gay”), while the perception of gay neighborhoods as relevant and meaningful began to fracture. As gay neighborhoods appear to “ decline” through LGBTQ + population loss (Spring 2021) and in other ways (Bitterman 2020, Hess 2019), new gay districts arise in processes of household migration and demographic shifts—spread across metropolitan space—in a pattern of succession and replacement (Doan and Atalay 2021; Bitterman 2021; Podmore 2021). Displaced LGBTQ + residents often re-group in other nearby locations—a sort of LGBTQ+  diaspora masked by mainstream integration—planting the seeds for the potential genesis of future gay neighborhoods (Bitterman 2021). Gentrification, shifting generational attitudes and social values, increasing use of technology and pandemic are among the many factors that influence the relevance and desirability of gay neighborhoods. Perhaps gentrification (and in some cases, hypergentrification) has run its course. In the early days of the COVID-19 pandemic, affluent people fled cites and urban neighborhoods in favor of greater space and isolation from others. As the gentrifiers move out, “queerdos” have begun to return to cities to reclaim their place (Kane 2020), resulting in new types of neighborhood forms and dynamics. This phenomenon calls into question whether the plateau at which we currently find gay neighborhoods portends the beginning of the end of gay neighborhoods, or the beginning of a new cycle for gay neighborhoods or simply part of the evolutionary process. Moreover, this plateau largely relates to gay neighborhoods in the Global North. Perhaps gay neighborhoods of a different or alternative sort may emerge as civil rights, recognition, and tolerance shifts across the Global South, evident in countries like India and the Philippines.

LGBTQ+ people migrate to new districts when they find safe, inclusive, and convenient access to everyday services and amenities—especially LGBTQ-friendly businesses and services—and now, perhaps more so than before 1990, the presence of services that support LGBTQ + families including schools, libraries, childcare centers, and family healthcare facilities. Gay neighborhoods appear to be at the vanguard edge of continual evolution—embodying a type of urban diaspora or metamorphosis—further evolving and adapting as LGTBQ+ individual and families re-sort themselves into new spaces (Andersson 2009). These “seed communities”—formative pockets that are too small yet to be considered proper neighborhoods—are the likely genesis points for future gay settlements that will emerge over time. These LGBTQ + micro-districts surface in expected places and unexpected places; the Hayes Valley in San Francisco, built partly on reclaimed urban land where a freeway was removed, is not exactly a gay neighborhood, but a gay-inclusive place populated by and visited by people connected with the famed Castro. A similar phenomenon is occurring in the Roncesvalles and Parkdale neighborhoods of Toronto, with LGBTQ + people migrating from Toronto’s legendary Church Street gayborhoods find places that proudly and outwardly welcome LGBTQ + individuals (Bitterman 2021). The potential reconfiguration of LGBTQ+  communities (physical communities, virtual communities , and other communities) is in opposition to an assumption in 2020 of urban decline following the COVID-19 pandemic and the stresses—economic decline, joblessness, a feared urban exodus, feelings of despair—it has caused (Batty 2020; Florida et al. 2020).

Perhaps “second generation” gay neighborhoods will serve future generations of LGBTQ + residents, citizens, families, and visitors by providing similar (and perhaps new, unimagined) functions just as established gay neighborhoods have served past generations (Bitterman and Hess 2021). While not all “seed” communities will flourish and some may even be extinguished by external forces, it is likely that as the needs of LGBTQ+ citizens and families change, so too will the types of neighborhoods these citizens and families require as gay neighborhoods potentially reconfigure for the future. In this way, gay neighborhoods could reconstitute around the archetype reflecting their existence for the previous five decades or in a form that does not yet exist or we cannot yet imagine. Moreover, we anticipate that established gay neighborhoods will propagate via an “ afterglow” (Coffin 2021) as historically relevant sites become landmarked or memorialized (Miller and Bitterman 2021).

 Gay neighborhoods have proven themselves resilient to the AIDS/HIV pandemic, economic change, population loss, demographic change, gentrification, and other forces. Given the evidence offered by chapters in this book and the thematic takeaway messages enumerated in this chapter, we argue that we are not witness to the “death” or even the uncontrolled decline of gay neighborhoods; instead, we suggest that gay neighborhoods by 2020 have reached a state of maturity and have ascended to a plateau in which a decentralized LGBTQ + populace may provide the catalyst for new forms of community engagement, activism, and relevance. The chapters in this volume emphasize the pressing need for supporting safe, inclusive, productive neighborhoods for LGBTQ+ people.

## References

[CR1] Aldrich R (2004). Homosexuality and the city: an historical overview. Urban Stud.

[CR2] Andersson J (2009). East end localism and urban decay: Shoreditch’s re-emerging gay scene. London J.

[CR3] Batty M (2020) The Coronavirus crisis: what will the post-pandemic city look like? Environ Plann. Published 14 May 2020. 10.1177/2399808320926912

[CR4] Binnie J (2014). Relational comparison, queer urbanism and worlding cities. Geogr Compass.

[CR5] Binnie J, Valentine G (1999). Geographies of sexuality—a review of progress. Prog Hum Geogr.

[CR6] Bitterman A (2020). Rainbow diaspora: the emerging renaissance of gay neighbourhoods. Town Plan Rev.

[CR7] Bitterman A, Bitterman A, Hess DB (2021). The rainbow connection: a time-series study of rainbow flag display across nine Toronto neighborhoods. The life and afterlife of gay neighborhoods: renaissance and resurgence.

[CR8] Bitterman A, Hess DB (2021) Understanding generation gaps in LGBTQ+ communities: perspectives about gay neighborhoods among heteronormative and homonormative generational cohorts In: Bitterman A, Hess DB (eds) The life and afterlife of gay neighborhoods: renaissance and resurgence. Springer, Dordrecht, Netherlands, pp 309–340

[CR9] Bitterman A, Hess DB (2016). Gay ghettoes growing gray: transformation of gay urban districts across North America reflects generational change. J Am Cult.

[CR10] Bitterman A, Hess DB (2016b) Will gay and lesbian neighborhoods resurge? Trump–Pence era underscores importance of LGBT communities Washington Blade 8 December

[CR11] Black D, Gates G, Sanders S, Taylor L (2002). Why do gay men live in San Francisco?. J Urban Econ.

[CR13] Brown G, Browne K, Elmhirst R, Hutta S (2010). Sexualities in/of the global south. Geogr Compass.

[CR14] Bruce K (2016). Pride parades: how a parade changed the world.

[CR15] Carpiano RM, Kelly BC, Easterbrook A, Parsons JT (2011). Community and drug use among gay men: the role of neighborhoods and networks. J Health Soc Behav.

[CR16] Castells M (1983). The city and the grassroots: a cross-cultural theory of urban social movements.

[CR17] Chauncey G (2008) Gay New York: gender, urban culture, and the making of the gay male world, 1890–1940. Hachette UK

[CR18] Christafore D, Leguizamon S (2018). Is ‘gaytrification’ a real phenomenon?. Urban Aff Rev.

[CR19] CNN (2019) Buttigieg: I wouldn’t be running if I hadn’t come out. https://www.cnn.com/videos/politics/2019/04/23/mayor-pete-buttgieg-coming-out-anderson-cooper-town-hall-vpx.cnn. Accessed 29 June 2020

[CR20] Coffin J, Bitterman A, Hess DB (2021). Plateaus and afterglows: theorizing the afterlives of gayborhoods as post-places. The life and afterlife of gay neighborhoods: renaissance and resurgence.

[CR21] Contreras EA (2019). Latinos and the liberal city: politics and protest in San Francisco.

[CR22] Crawford-Lackey K, Springate ME (2020). Communities and place: a thematic approach to the histories of LGBTQ communities in the United States.

[CR23] Doan P (2007). Queers in the American City: transgendered perceptions of urban space. Gend Place Cult.

[CR24] Doan P (2015). Planning and LGBTQ communities: the need for inclusive queer spaces.

[CR25] Doan P, Higgins H (2011). The demise of queer space? Resurgent gentrification and the assimilation of LGBT neighborhoods. J Plan Educ Res.

[CR26] Doan P, Atalay O, Bitterman A, Hess DB (2021). After the life of LGBTQ spaces: Learning from Atlanta and Istanbul. The life and afterlife of gay neighborhoods: renaissance and resurgence.

[CR27] Duberman M (1991) About time: exploring the gay past. Plume Books

[CR28] Duberman M (2018) Has the gay movement failed? University of California Press

[CR29] Eeckhout B, Herreman B, Dhoest A, Bitterman A, Hess DB (2021). A gay neighbourhood or merely a temporary cluster of “strange” bars? Gay bar culture in Antwerp. The life and afterlife of gay neighborhoods: renaissance and resurgence.

[CR30] Elledge J (2018). The boys of fairy town: sodomites, female impersonators, third-sexers, pansies, queers, and sex morons in Chicago’s first century.

[CR31] Evanosky D, Kos EJ, Mondon K (2018). San Francisco: then and now.

[CR32] Florida R (2004). The rise of the creative class and how it’s transforming work, leisure, community and everyday life.

[CR33] Florida R, et al (2020) How life in our cities will look after the coronavirus pandemic. Foreign Policy. https://foreignpolicy.com/2020/05/01/future-of-cities-urban-life-after-coronavirus-pandemic/. Accessed 10 September 2020

[CR34] Forstie C (2008). Theory making from the middle: researching LGBTQ communities in small cities. City Community.

[CR35] Forsyth A (2001). Sexuality and space: nonconformist populations and planning practice. J Plan Lit.

[CR36] Frisch M, Bitterman A, Hess DB (2021). A queer reading of the United States census. The life and afterlife of gay neighborhoods: renaissance and resurgence.

[CR37] Gates G, Ost J (2004). The gay & lesbian atlas. The Urban Institute. Washington, DC

[CR38] Ghaziani A (2014). Measuring urban sexual cultures. Theor Soc.

[CR39] Ghaziani A (2015a) There goes the gayborhood? Princeton University Press

[CR40] Ghaziani A (2015). Gay enclaves face prospect of being passé: how assimilation affects the spatial expressions of sexuality in the United States. Int J Urban Reg Res.

[CR41] Ghaziani A, Bitterman A, Hess DB (2021). Why gayborhoods matter: the street empirics of urban sexualities. The life and afterlife of gay neighborhoods: renaissance and resurgence.

[CR42] Gieseking J (2020) A queer New York: geographies of lesbians, dykes, and queers. New York University Press

[CR43] Giraud C (2014). Gay neighborhoods/quartiers gays.

[CR44] Gorman-Murray A, Nash CJ (2016). Transformations in LGBT consumer landscapes and leisure spaces in the neoliberal city. Urban Stud.

[CR45] Gorman-Murray A, Nash CJ, Bitterman A, Hess DB (2021). recovering the gay village: a comparative historical geography of urban change and planning in Toronto and Sydney. The life and afterlife of gay neighborhoods: renaissance and resurgence.

[CR46] Hemmings C (2002). Bisexual spaces: a geography of sexuality and gender.

[CR47] Hess DB (2019). Effects of gentrification and real-estate market escalation on gay neighbourhoods. Town Plan Rev.

[CR48] Higgs D (ed) (1999) Queer sites: gay urban histories since 1600. Routledge, New York

[CR49] Jacobs J (1961). The death and life of great American cities.

[CR50] Kane PL (2020) ‘Rich people leave, artists and queerdos return’: is San Francisco’s tech exodus real or a fantasy? The Guardian, September 12. https://www.theguardian.com/us-news/2020/sep/12/san-francisco-tech-workers-leaving-rent-covid-remote-work. Accessed 12 Sept 2020

[CR51] Khubchandani K (2020). Ishtyle: accenting gay Indian nightlife.

[CR52] Lauria M, Knopp L (1985). Toward an analysis of the role of gay communities in the urban renaissance. Urban Geogr.

[CR53] Levine MP (1979). Gay ghetto. J Homosex.

[CR54] Martel F, Bronski M, Baudoin P, Martel F (2018). Global gay: how gay culture is changing the world.

[CR55] Mattson G (2019). Are gay bars closing? using business listings to infer rates of gay bar closure in the United States, 1977–2019. Socius.

[CR56] McPherson M, Smith-Lovin L, Cook JM (2001). Birds of a feather: homophily in social networks. Ann Rev Sociol.

[CR57] Miles S, Bitterman A, Hess DB (2021). Let’s (not) go outside: Grindr, hybrid space, and digital queer neighbourhoods. The life and afterlife of gay neighborhoods: renaissance and resurgence.

[CR58] Miles S, Coffin J, Ghaziani A, Hess DB, Bitterman A, Bitterman A, Hess DB (2021). After/lives: insights from the COVID-19 pandemic for gay neighborhoods. The life and afterlife of gay neighborhoods: renaissance and resurgence.

[CR59] Miller C, Bitterman A, Bitterman A, Hess DB (2021). Commemorating historically significant gay places across the United States. The Life And Afterlife Of Gay Neighborhoods: Renaissance And Resurgence.

[CR60] Moss J (2017) Vanishing New York: how a great city lost its soul. HarperCollins

[CR61] Murray TL (2014). Gay neighborhoods: fourth Street’s “outing” and a comparison of homosexual geographies.

[CR62] Nagourney A (2019). PRIDE: fifty years of parades and protests from the photo archives of the New York Times.

[CR63] Nash CJ, Gorman-Murray A (2014). LGBT neighbourhoods and ‘new mobilities’: towards understanding transformations in sexual and gendered urban landscapes. Int J Urban Reg Res.

[CR64] Nash CJ, Gorman-Murray A (2015). Recovering the gay village: a comparative historical geography of urban change and planning in Toronto and Sydney. Hist Geogr.

[CR65] Nash CJ, Gorman-Murray A (2015). Lesbians in the city: mobilities and relational geographies. Journal of Lesbian Studies.

[CR66] Niedt G, Bitterman A, Hess DB (2021). A tale of three villages: contested discourses of place-making in central philadelphia. The life and afterlife of gay neighborhoods: renaissance and resurgence.

[CR67] Notaro S (2020). Marginality and global LGBT communities: conflicts, civil rights and controversy.

[CR68] Orne J (2017). Boystown: sex and community in Chicago.

[CR69] Podmore J (2001). Lesbians in the crowd: gender, sexuality and visibility along Montréal’s Boul St-Laurent. Gender, Place Cult.

[CR70] Podmore J, Bitterman A, Hess DB (2021). Far beyond the gay village: LGBTQ Urbanism and generation in Montréal’s mile end. The life and afterlife of gay neighborhoods: renaissance and resurgence.

[CR71] Potts L (2016). Faces of a neighborhood: Boston’s South End in the early twenty-first century.

[CR72] Ross B, Sullivan R (2012). Tracing lines of horizontal hostility: how sex workers and gay activists battled for space, voice, and belonging in Vancouver, 1975–1985. Sexualities.

[CR01] Ruting B (2008) Economic transformations of gay urban spaces: revisiting Collins’ evolutionary gay district model. Aust Geogr 39(3):259–269

[CR73] Ryan H (2020). When Brooklyn was queer.

[CR74] Seidman S (1994). Queer-ing sociology, sociologizing queer theory: an introduction. Sociol Theor.

[CR75] Seidman S (2004) Beyond the closet: the transformation of gay and lesbian life. Psychology Press

[CR76] Shaw R (2015). The Tenderloin: sex, crime, and resistance in the heart of San Francisco.

[CR77] Spring A, Bitterman A, Hess DB (2021). Breaking down segregation: shifting geographies of male same-sex households within desegregating cities. The life and afterlife of gay neighborhoods: renaissance and resurgence.

[CR78] Stone AL, Bitterman A, Hess DB (2021). Wearing pink in Fairy Town: the heterosexualization of the Spanish Town neighborhood and carnival parade in Baton Rouge. The life and afterlife of gay neighborhoods: renaissance and resurgence.

[CR79] Tongson K (2011) Relocations: Queer suburban imaginaries. New York University Press

[CR81] Weinke C, Whaley RB, Braatz R, Bitterman A, Hess DB (2021). Are “gay” and “queer-friendly” neighbourhoods healthy? Assessing how areas with high densities of same-sex couples impact the mental health of sexual minority and majority young adults. The life and afterlife of gay neighborhoods: renaissance and resurgence.

[CR82] Wolf DG (1979) The lesbian community. University of California Press

[CR83] Wu S, Ward J (2018). The mediation of gay men’s lives: a review on gay dating app studies. Sociol Compass.

[CR84] Zukin S (1998). Urban lifestyles: diversity and standardisation in spaces of consumption. Urban Stud.

